# Comparative Analysis of Neonatal Effects in Pregnant Women with Cardiovascular Risk versus Low-Risk Pregnant Women

**DOI:** 10.3390/jcm12124082

**Published:** 2023-06-16

**Authors:** Simona-Alina Abu-Awwad, Marius Craina, Adrian Gluhovschi, Lioara Boscu, Elena Bernad, Mircea Iurciuc, Ahmed Abu-Awwad, Stela Iurciuc, Cristina Tudoran, Robert Bernad, Anca Laura Maghiari

**Affiliations:** 1 Doctoral School, “Victor Babes” University of Medicine and Pharmacy, Eftimie Murgu Square, No. 2, 300041 Timisoara, Romania; alina.abuawwad@umft.ro; 2 “Clinic of Obstetrics and Gynecology”, “Pius Brinzeu” County Clinical Emergency Hospital, 300723 Timisoara, Romania; mariuscraina@hotmail.com (M.C.); adigluhovschi@yahoo.com (A.G.); ebernad@yahoo.com (E.B.); boscu.anca@umft.ro (A.L.M.); 3 Department of Obstetrics and Gynecology, Faculty of Medicine, “Victor Babes” University of Medicine and Pharmacy, 300041 Timisoara, Romania; 4 Center for Laparoscopy, Laparoscopic Surgery and In Vitro Fertilization, “Victor Babes” University of Medicine and Pharmacy, 300041 Timisoara, Romania; 5 Senate Office, “Victor Babes” University of Medicine and Pharmacy, Eftimie Murgu Square, No. 2, 300041 Timisoara, Romania; lioara.boscu@umft.ro (L.B.); mirceaiurciuc@gmail.com (M.I.); iurciuc.stela@umft.ro (S.I.); 6 Departament VI—Discipline of Outpatient Internal Medicine, Cardiovascular Prevention and Recovery, “Victor Babes” University of Medicine and Pharmacy, Eftimie Murgu Square, No. 2, 300041 Timisoara, Romania; 7 Department XV—Discipline of Orthopedics—Traumatology, “Victor Babes” University of Medicine and Pharmacy, Eftimie Murgu Square, No. 2, 300041 Timisoara, Romania; 8 Research Center University Professor Doctor Teodor Șora, “Victor Babes” University of Medicine and Pharmacy, Eftimie Murgu Square, No. 2, 300041 Timisoara, Romania; 9 Discipline of Cardiology, “Victor Babes” University of Medicine and Pharmacy, Eftimie Murgu Square, No. 2, 300041 Timisoara, Romania; cristina13.tudoran@gmail.com; 10 “Pius Brinzeu” Emergency County Clinical Hospital, Blvd Liviu Rebreanu, No. 156, 300723 Timisoara, Romania; 11 Department of Automatic Control and Applied Informatics, Politehnica University of Timisoara, 300223 Timisoara, Romania; robibernad11@gmail.com; 12 Department I—Discipline of Anatomy and Embryology, “Victor Babes” University of Medicine and Pharmacy, Eftimie Murgu Square, No. 2, 300041 Timisoara, Romania

**Keywords:** pregnancy, pregnant woman, high risk, pathology, cardiovascular disease, neonatal effects, fetal development

## Abstract

Background: Cardiovascular diseases are a leading cause of mortality and morbidity worldwide. Pregnancy imposes unique physiological changes on a woman’s cardiovascular system. Materials and Methods: A cohort of 68 participants, comprising 30 pregnant women with cardiovascular risk and 38 without cardiovascular risk, was recruited for this study. These participants were prospectively followed during their pregnancies from 2020 to 2022 at the Obstetrics and Gynecology Department of the “Pius Brînzeu” Emergency County Clinical Hospital in Timişoara, Romania. All women included in this study underwent cesarean section deliveries at the same medical facility. Data regarding the gestational weeks at delivery, birth weight, and Apgar scores assessed by neonatologists were collected for each participant. Statistical analyses were performed to compare the neonatal effects between the two groups. Results: The results of this study revealed significant differences between the groups in terms of Apgar scores (*p* = 0.0055), gestational weeks (*p* = 0.0471), and baby birth weight (*p* = 0.0392). Conclusion: The findings underscore the importance of considering maternal cardiovascular health as a potential determinant of neonatal outcomes. Further research is needed to elucidate the underlying mechanisms and develop strategies for optimizing neonatal outcomes in high-risk pregnancies.

## 1. Introduction

Women worldwide experience cardiovascular diseases as the primary reasons for illness and death, highlighting their significance in terms of morbidity and mortality. The physiological foundation of cardiovascular well-being differs between men and women, resulting in diverse cardiovascular reactions to stimuli and the manifestation of symptoms related to cardiovascular diseases. These variations directly impact the effectiveness of treatments and outcomes [1]. Pregnancy, with its physiological changes and increased cardiovascular demands, poses additional challenges for women with pre-existing cardiovascular risk factors. It is well-established that maternal cardiovascular health plays a crucial role in the well-being of both the mother and the developing fetus [2]. The impact of cardiovascular risk factors on maternal and fetal outcomes has been extensively studied but there remains a need for further investigation, particularly regarding neonatal effects.

The neonatal period is a critical time for assessing the well-being and overall health of newborns. The Apgar score, developed by Dr. Virginia Apgar in the 1950s [3], is a widely used tool implemented to evaluate the newborn’s condition immediately after birth. It provides a standardized assessment of vital signs, including heart rate, respiratory effort, muscle tone, reflex irritability, and color. The Apgar score offers valuable insights into the neonate’s transition to extrauterine life and serves as an initial assessment of their overall well-being [4].

While the Apgar score is routinely used to assess neonatal health, its association with maternal cardiovascular risk factors during pregnancy remains an area of active research [5]. Understanding how cardiovascular risk factors in pregnant women may influence neonatal outcomes can help healthcare providers identify high-risk pregnancies and implement appropriate interventions to optimize neonatal health.

Therefore, this study aims to conduct a comparative analysis of neonatal effects in pregnant women with cardiovascular risk factors versus low-risk pregnant women. By evaluating the Apgar scores of newborns from both groups, we can gain insights into the potential impact of maternal cardiovascular risk factors on neonatal well-being.

We hypothesize that pregnant women with cardiovascular risk factors will have infants with lower Apgar scores compared to low-risk pregnant women. This hypothesis is based on the premise that compromised maternal cardiovascular health may lead to suboptimal fetal development and neonatal outcomes [6].

Maternal cardiovascular health plays a crucial role in fetal development and can influence the growth and overall health of the baby [7]. Several factors related to maternal cardiovascular risk, such as hypertension [8], diabetes [9], obesity [10], and smoking [11], have been associated with adverse effects on birth weight. Some of these can lead to low birth weight while others can result in macrosomia.

Hypertensive disorders of pregnancy, such as preeclampsia, can also impact birth weight [12]. Preeclampsia is characterized by high blood pressure and damage being caused to organs, including the placenta. It can lead to an inadequate blood supply to the fetus, contributing to low birth weight [13].

Maternal cardiovascular health can also influence the duration of gestation and the risk of preterm birth [14]. Preterm birth occurs when a baby is born before 37 completed weeks of gestation [15]. These conditions can disrupt the normal physiological processes of pregnancy, leading to complications that necessitate the early delivery of the baby.

It is important to note that while there is a link between maternal cardiovascular risk and the number of gestational weeks, many other factors can also contribute to the length of a pregnancy. Proper management of cardiovascular health during pregnancy is crucial to minimize the risk of complications and ensure optimal gestational duration for the well-being of both the mother and the baby.

This study aimed to compare neonatal effects in pregnant women with cardiovascular risk factors to those of low-risk pregnant women, focusing on Apgar score, birth weight, and delivery gestational weeks. The findings of this comparative analysis shed light on the potential impact of cardiovascular risk factors on neonatal outcomes, providing valuable insights for prenatal care and management.

This study’s findings have the potential to contribute valuable information to clinical practice, assisting healthcare providers in identifying and managing high-risk pregnancies more effectively. Additionally, it may highlight the importance of addressing maternal cardiovascular risk factors to improve neonatal health outcomes.

## 2. Materials and Methods

This study was conducted at the Department of Obstetrics and Gynecology in the ‘Pius Brinzeu’ County Emergency Clinical Hospital in Timisoara, Romania, spanning from 1 January 2020 to 31 December 2022. The research population and their relevant characteristics were identified using a population-based administrative database of patients who received outpatient care at the same hospital throughout the study period. The medical records of the patients were securely stored in a database that adhered to privacy laws; access to these records was obtained with the patients’ consent.

The database contained comprehensive information, including demographic data, medical history, and the in-hospital procedures of the patients. The baseline characteristics and procedures of all patients were recorded both in the hospital database and in paper patient records, which were thoroughly reviewed by certified clinicians involved in this study. The implementation of an interconnected database that maintains patient information regardless of their location is of the utmost importance.

For this study, we obtained ethical approval (No. 265/22 September 2022) from the management of the “Pius Brînzeu” County Emergency Hospital in Timișoara. This approval serves as a testament to our commitment to conducting the research in an ethical manner and ensures the protection of the participants’ rights and well-being throughout this study.

By comparing the neonatal effects between these two groups, this study aims to shed light on the potential impact of cardiovascular risk factors on the well-being of newborns. The findings from this research may have significant implications for clinical practice and contribute to improving the care and management of pregnant women with cardiovascular risk factors, ultimately leading to better neonatal outcomes.

Inclusion criteria for this study:

○ Women who have given birth in the second or third trimester of pregnancy;
○ Pregnant women over the age of 18;
○ Women who have received prenatal care and had regular check-ups throughout their pregnancy;
○ Women who have given their informed consent to be included in this study;
○ Having no record of a positive COVID-19 diagnosis within the past year.

This study excluded participants who met the following exclusion criteria:

○ Women with a previous history of substance abuse or alcohol dependency;
○ Women with a prior history of psychiatric disorders or mental health conditions;
○ Women with a previous medical history of thromboembolic disease or disorders affecting blood clotting;
○ Women who have been involved in alternative clinical trials or studies in the preceding 3 months;
○ Women who have experienced negative responses to blood collection or phlebotomy procedures in the past;
○ Women who are incapable or uninterested in providing informed consent to take part in this study;
○ Patients who have been diagnosed with infectious conditions, such as hepatitis B or C virus (HBV, HCV) and human immunodeficiency virus (HIV), as well as those who have acquired immunodeficiency syndrome (AIDS);
○ Patients with inadequately managed metabolic disorders;
○ Patients with inadequately managed endocrine disorders.

In the group of patients with cardiovascular risk or cardiovascular disease, the patients who met at least one of the following criteria were included:

○ Smoking history exceeding 5 years;
○ Presence of cardiovascular disease in the family;
○ Limited engagement in physical activity or leading a predominantly sedentary lifestyle;
○ Dietary pattern characterized by excessive consumption of saturated and trans fats, sodium, and added sugars, contributing to an overall unhealthy nutritional profile;
○ Sleep apnea/other sleep disorders;
○ Preexisting hypertension in pregnancy;
○ Pregnancy-induced hypertension;
○ Preeclampsia;
○ Eclampsia;
○ Low-density lipoprotein cholesterol over 190 mg/dL;
○ Total cholesterol over 280 mg/dL;
○ Triglycerides over 200 mg/dL.

In Table 1, we have defined the cardiovascular pathologies present in this study.

Sleep disorders are an inclusive classification encompassing a range of irregularities that occur during sleep, such as snoring, obstructive sleep apnea (OSA), central sleep apnea (CSA), respiratory-related arousals, and hypoventilation [20].

For each participant, we recorded the gestational weeks at the time of delivery, birth weight, and Apgar scores, as evaluated by the neonatologist.

Based on gestational age, we identified patients who delivered preterm. According to the World Health Organization (WHO), preterm birth is defined as the delivery of a baby before completing 37 weeks of gestation or fewer than 259 days from the first date of a woman’s last menstrual period (LMP) [21]. Based on birth weight, we identified patients who gave birth to infants with intrauterine growth restriction (IUGR). Intrauterine growth restriction (IUGR) refers to a condition in which a fetus fails to reach its expected growth potential while in the womb. It is characterized by a slower rate of growth compared to what is typically observed for gestational age [22].

The data collected in this study were analyzed using GraphPad Prism (version 5). Prior to analysis, the data underwent screening for outliers, missing values, and normality. If necessary, variables were transformed or normalized to meet the assumptions of the statistical tests.

Descriptive statistics were utilized to provide an overview of the data, while group differences were evaluated using *t*-tests. The *t*-test is a statistical technique commonly applied when dealing with small sample sizes to determine whether there is a significant difference between the means of two groups. By comparing the means of the two groups, the *t*-test determines whether the observed difference is statistically significant or simply a result of random chance.

All statistical tests conducted were two-tailed and *p*-values lower than 0.05 were considered statistically significant. The results are reported as mean ± standard deviation (SD). The mean, also referred to as the average, is a statistical measure representing the central tendency of a dataset. It is calculated by summing all the values in the dataset and dividing the total by the number of values. The standard deviation is a statistical measure indicating the extent of variability or dispersion in a dataset. It provides information on the spread or variation of data points around the mean or average value. Graphs were generated using GraphPad Prism, with appropriate labeling and formatting to ensure a clear presentation.

## 3. Results

This study included a total of sixty-eight pregnant women who underwent delivery through cesarean section (C-section) at the same medical institution. A cesarean section is a surgical procedure in which a baby is delivered through an incision made in the mother’s abdomen and uterus. It is typically performed when a vaginal birth is not feasible or safe for either the mother or the baby.

The participants were divided into two groups: Group 1 consisted of thirty-eight pregnant women without cardiovascular risk or cardiovascular disease; meanwhile, Group 2 included thirty pregnant women with cardiovascular risk or cardiovascular disease. In the context of this study, cardiovascular risk refers to the presence of risk factors or pre-existing conditions that increase the likelihood of developing cardiovascular disease during or after pregnancy.

Table 2 presents the distribution of patients from Group 2 categorized based on their cardiovascular risk.

Table 3 provides an overview of how participants from both groups are distributed across different age categories. The age categories are determined based on specific criteria or ranges of age. By examining this table, we can observe the proportion of participants in each age category within each group.

Since the age differences are not statistically significant, this study results are not influenced by age categories.

Table 4 presents the results for the Apgar scores, gestational weeks, and baby birth weight, along with relevant statistical measures. The participants were divided into two groups: Group 1 consisted of thirty-eight pregnant women without cardiovascular risk or cardiovascular disease; meanwhile, Group 2 included thirty pregnant women with cardiovascular risk or cardiovascular disease. In the context of this study, cardiovascular risk refers to the presence of risk factors or pre-existing conditions that increase the likelihood of developing cardiovascular disease during or after pregnancy.

For each variable, the following statistical measures are provided: minimum (the smallest recorded value for the respective variable), 25th percentile (the value below which 25% of the observed values fall, also known as the first quartile), median (the value that divides the distribution into two equal halves, with 50% of the observed values below and 50% above), 75th percentile (the value below which 75% of the observed values fall, also known as the third quartile), maximum (the largest recorded value for the respective variable), mean (the average value calculated by summing all of the values and dividing by the total number of observations), and standard deviation (a statistical measure indicating the extent of variability or dispersion around the mean value).

*p*-value *t*-test: For the variable “Gestational weeks,” the *p*-value indicates the statistical significance of any observed differences between groups.

In terms of those from Group 1, five patients gave birth prematurely and just two patients gave birth to infants with IUGR. Regarding those from Group 2, ten patients gave birth prematurely and six patients gave birth to infants with IUGR. These results are presented in Table 5.

## 4. Discussion

The comparative analysis of neonatal effects in pregnant women with cardiovascular risk versus low-risk pregnant women provides valuable insights into the potential impact of cardiovascular health on neonatal outcomes. Our study aimed to evaluate and compare various neonatal parameters between these two groups.

Offspring born to pregnant women with cardiovascular diseases or cardiovascular risks had elevated rates of neonatal complications. Increased risks of NACE (neonatal adverse cardiac events) were independently linked to preeclampsia, significant adverse cardiac events, obstetric complications, and preexisting diabetes mellitus [23].

The findings of this study demonstrated significant differences in neonatal outcomes between the two groups. Pregnant women with cardiovascular risk factors were more likely to experience adverse neonatal effects compared to low-risk pregnant women [24].

In this study, we conducted a comparative analysis of the neonatal effects between pregnant women with cardiovascular risk factors and low-risk pregnant women. Our analysis focused on evaluating the Apgar scores, infant birth weight, and gestational weeks at delivery as important indicators of neonatal well-being.

The Apgar score is a widely used tool to assess the overall health and well-being of newborns immediately after birth. It provides a quick assessment of the infant’s heart rate, respiratory effort, muscle tone, reflex irritability, and color [25]. Our results revealed that neonates born to pregnant women with cardiovascular risk factors had slightly lower Apgar scores compared to neonates born to low-risk pregnant women.

Another important aspect we investigated was infant birth weight. Birth weight is a critical factor in assessing the overall health and development of newborns. A higher incidence of neonatal mortality and negative outcomes among infants who survive is linked to lower birth weight [26]. We observed that infants born to pregnant women with cardiovascular risk factors tended to have slightly lower birth weights compared to those born to low-risk pregnant women. Although this difference was statistically significant, the magnitude of the difference was relatively small and may not have significant clinical implications.

Furthermore, we examined gestational weeks at delivery as a measure of fetal development and maturity. Premature birth can also be a contributing factor to hypertensive disorders during pregnancy. Preterm birth is associated with various adverse outcomes; understanding the potential influence of cardiovascular risk factors on gestational age is crucial. Approximately 75% of perinatal mortality is attributed to preterm births and these premature infants also experience more than half of the long-term morbidity. While the majority of preterm babies survive, they face higher risks of neurodevelopmental impairments, as well as respiratory and gastrointestinal complications [27].

The association between cardiovascular risk factors and adverse neonatal outcomes can be attributed to various factors. Poor cardiovascular health in pregnant women may lead to compromised placental function [28], inadequate fetal oxygenation [29], and poor nutrient supply [30]. These physiological changes can contribute to impaired fetal growth and development, resulting in adverse neonatal outcomes.

Our study highlights the importance of early identification and management of cardiovascular risk factors during pregnancy. Healthcare providers should prioritize regular monitoring and appropriate interventions for pregnant women with pre-existing cardiovascular conditions or those at risk for developing cardiovascular complications. This may involve close collaboration between obstetricians, cardiologists, and other specialists to optimize maternal cardiovascular health and improve neonatal outcomes.

The rationale for including only patients who underwent cesarean delivery in this article can be attributed to several factors.

Firstly, cesarean delivery is a surgical procedure that allows for controlled and standardized conditions during childbirth. This ensures consistency in data collection and reduces potential confounding variables that may arise from the variability in vaginal deliveries.

Secondly, the focus of this study appears to be on comparing the neonatal effects between pregnant women with cardiovascular risk and low-risk pregnant women. By restricting the sample to cesarean deliveries, the researchers can more effectively analyze and attribute any observed differences in neonatal outcomes to the underlying cardiovascular risk factor, independent of the mode of delivery.

Furthermore, cesarean deliveries are often recommended or preferred in certain high-risk pregnancies, such as those with maternal cardiovascular conditions, to mitigate potential complications or risks associated with vaginal delivery. Thus, by including only cesarean deliveries, this study can specifically investigate the impact of cardiovascular risk on neonatal outcomes while minimizing confounding factors related to the mode of delivery.

However, it is important to acknowledge that this selection criterion may limit the generalizability of the findings to populations where vaginal deliveries are more prevalent. Transparency regarding the rationale for the selection of cesarean deliveries in this study should be provided, along with a discussion of the potential implications and limitations of this approach in the interpretation of the results.

### Strengths and Limitations

One of the major strengths is that this study has a well-defined design, focusing on two distinct groups based on cardiovascular risk status. This allows for a direct comparison between the two groups and facilitates the examination of potential differences in neonatal outcomes.

Another strength is that this study examines crucial neonatal outcomes, including Apgar scores, birth weight, and gestational weeks at delivery. These outcomes are widely recognized as essential indicators of neonatal health and well-being, ensuring this study’s relevance and clinical significance.

By comparing neonatal outcomes between pregnant patients with and without cardiovascular risk factors, this study has the potential to provide valuable insights into the impact of cardiovascular risk on neonatal health. This information can contribute to improving prenatal care and management of pregnant women with cardiovascular risk.

One of the limitations is that this study’s findings may not be generalizable to the entire population of pregnant women due to the potential for selection bias. This study was conducted in a single hospital, which may limit the diversity of the patient population and introduce biases related to the specific characteristics of the hospital’s patient population.

Another limitation is that, despite efforts to match the two groups based on cardiovascular risk, there may be other confounding factors that were not adequately controlled for. Factors, such as socioeconomic status, ethnicity, or comorbidities, could impact neonatal outcomes but were not addressed in this study design.

This study’s focus on immediate neonatal outcomes, such as Apgar scores, birth weight, and gestational weeks at delivery, may provide limited insights into the long-term effects of cardiovascular risk on neonatal health and development. Long-term follow-up studies would be necessary to comprehensively evaluate the impact of cardiovascular risk on outcomes beyond the immediate postnatal period.

Further research is needed to explore the underlying mechanisms linking cardiovascular risk factors and adverse neonatal outcomes. Prospective studies with larger sample sizes and diverse populations would help confirm and expand upon our findings. Additionally, investigating the long-term implications of neonatal effects in these populations is warranted.

## 5. Conclusions

The comparative analysis of neonatal effects in pregnant women with cardiovascular risk versus low-risk pregnant women reveals important implications for clinical practice and patient care. Our study demonstrates that pregnant women with cardiovascular risk factors are at a higher risk of adverse neonatal outcomes compared to low-risk pregnant women. These outcomes include preterm birth, lower Apgar scores, and increased rates of neonatal complications.

The findings emphasize the need for proactive identification and the management of cardiovascular risk factors during pregnancy. Healthcare providers should prioritize regular monitoring and appropriate interventions to optimize maternal cardiovascular health and improve neonatal outcomes. This may involve multidisciplinary collaboration between obstetricians, cardiologists, and other specialists to ensure comprehensive care for high-risk pregnant women.

It is important to mention that the presence of cardiovascular risk factors with a negative impact on pregnancy can also affect the mother’s physical well-being. Therefore, it is crucial to identify and properly treat these factors for optimal maternal health. The perinatal period, which encompasses pregnancy and the postpartum period, is a vulnerable time for women’s mental health. While postpartum depression has received significant attention, the association between personality dimensions, trait anxiety, and suicide ideation during the perinatal period remains relatively unexplored. Understanding the psychological factors contributing to suicide ideation in this specific population is crucial for identifying at-risk individuals and developing effective interventions [31].

Further research is warranted to delve into the underlying mechanisms linking cardiovascular risk factors and adverse neonatal outcomes. Prospective studies with larger sample sizes and diverse populations will provide more robust evidence and enable the development of targeted interventions.

In conclusion, this study underscores the importance of addressing cardiovascular risk factors in pregnant women to minimize adverse neonatal effects. By focusing on risk identification, monitoring, and intervention, healthcare professionals can improve the well-being and long-term outcomes of both mothers and their newborns.

## Figures and Tables

**Table 1:** Definition of cardiovascular pathologies present in this study.

Term/Condition	Definition
Preexisting Hypertension In Pregnancy/Chronic Hypertension	Blood pressure ≥ 140/90 mmHg before pregnancy or before the 20th week of gestation [16].
Pregnancy-Induced Hypertension	It is classified as having gestational hypertension when a woman, who previously had normal blood pressure, experiences a systolic blood pressure equal to or higher than 140 mmHg, a diastolic blood pressure equal to or higher than 90 mmHg, or both on two separate occasions at least 4 hours apart after reaching the 20th week of pregnancy [17].
Preeclampsia	A pregnancy complication characterized by the onset of high blood pressure, typically occurring after 20 weeks of gestation and commonly near the end of pregnancy. This condition may or may not be accompanied by the appearance of protein in the urine [18].
Eclampsia	Seizures or convulsions in a woman who previously had pregnancy-induced hypertension, such as gestational hypertension or preeclampsia [19].
Obstructive Sleep Apnea	OSA (Obstructive Sleep Apnea) is distinguished by recurrent episodes of upper airway collapse during sleep, which are accompanied by repetitive declines in oxygen levels, frequent awakenings, and fragmented sleep patterns. Affected individuals may exhibit typical symptoms, such as snoring, inadequate sleep quality, awakenings marked by choking sensations, diminished neurocognitive performance, and in severe cases, the potential for motor vehicle accidents resulting from brief lapses of consciousness referred to as “micro-sleeps” while driving [20].

**Table 2:** Distribution of patients from Group 2 (women with cardiovascular risk or cardiovascular disease) categorized according to their cardiovascular risk.

Maternal Cardiovascular Risk	Group 2 (*n* = 40)
○ >5 years smoking patients	56.6%
○ Family history of cardiovascular disease	41%
○ Sleep apnea or other sleep disorders	3.34%
○ Preexisting hypertension in pregnancy	10%
○ Pregnancy-induced hypertension	26.8%
○ Preeclampsia	11%
○ Eclampsia	6.66%
○ Low-density lipoprotein cholesterol > 190 mg/dL	72%
○ Total cholesterol > 280 mg/dL	63.3%
○ Triglycerides > 200 mg/dL	83.4%

**Table 3:** Distribution of patients from Group 1 (women without cardiovascular risk or cardiovascular disease) and Group 2 (women with cardiovascular risk or cardiovascular disease) categorized according to their age categories.

	Group 1 (*n* = 48)	Group 2 (*n* = 40)	*p* Value
Age between 18–29 years old	47.36%	43.3%	0.8998
Age between 30–40 years old	52.63%	56.6%	0.9831

**Table 4:** Comparison of Apgar scores, gestational weeks, and baby birth weight between Group 1 (women without cardiovascular risk/cardiovascular disease) and Group 2 (women with cardiovascular risk/cardiovascular disease).

	Apgar Scores		Gestational Weeks		Baby Birth Weight	
	Group 1 (*n* = 48)	Group 2 (*n* = 40)	Group 1 (*n* = 48)	Group 2 (*n* = 40)	Group 1 (*n* = 48)	Group 2 (*n* = 40)
Minimum	7	7	35	28	2490	1300
25% Percentile	9	8	38	37.25	2960	2858
Median	9	9	38	38	3370	3195
75% Percentile	9	9	39	39	3650	3490
Maximum	10	9	41	41	4400	4400
Mean	8.938	8.550	38.38	37.75	3363	3118
Std. Deviation	0.5981	0.6775	1.024	2.239	456.5	629.8
*p*-value *t*-test	0.0055		0.0471		0.0392	

**Table 5:** Preterm Birth and Infant Growth Restriction (IUGR) Frequencies in Two Patient Groups.

	Women without Cardiovascular Risk/Cardiovascular Disease	Women with Cardiovascular Risk/Cardiovascular Disease
Preterm Birth	10%	25%
Term Birth	90%	75%
Normal Weight Infants	96%	85%
Infants With Iugr	4%	15%
